# 1777. Shorter Versus Longer Antibiotic Durations for Coagulase-Negative Staphylococcus Catheter-Related Bloodstream Infections and Potential Impact on Antimicrobial Stewardship in a County Hospital

**DOI:** 10.1093/ofid/ofac492.1407

**Published:** 2022-12-15

**Authors:** Niki Arab, Oscar E Gallardo Huizar, Elizabeth Aquije, Marsha R Cheng, Brian Kim, Joseph Isaac S Wong, Arthur Jeng

**Affiliations:** Olive View- UCLA Medical Center, Los Angeles, California; Olive View-UCLA, Los Angeles, California; Olive View Medical Center, Jacksonville, Florida; Olive View-UCLA Medical Center, Los Angeles, California; Olive View-UCLA Medical Center, Los Angeles, California; Olive View UCLA Medical Center, Arcadia, California; Olive View UCLA Medical Center/UCLA School of Medicine, Sylmar, California

## Abstract

**Background:**

Coagulase negative Staphylococcus (CoNS) is a common cause of catheter-related bloodstream infection (CRBSI). Current guidelines are to provide 10-14 days (d) of antibiotics (abx) plus lock abx in the absence of catheter removal, and if the catheter has been removed, 5-7 d after removal. Olive View UCLA-Medical Center (Sylmar, CA) is an academic county hospital, serving the medical needs of the underserved community. The purpose of this study was to evaluate differences in treatment (tx) durations for CoNS CRBSI on clinical outcomes and evaluate the potential impact on antimicrobial stewardship.

**Methods:**

Single center retrospective chart review from January 2018 to December 2021 on all central line-obtained blood cultures (Bcx) positive for CoNS. Exclusion criteria included suspected contamination or no abx tx, invasive CoNS infection, and concomitant infection treated with abx that have activity against CoNS. Duration of abx tx was divided into two groups: >10 d vs ≤10 d with or without catheter removal and >7 d vs ≤7 d post catheter removal. Primary outcomes assessed were recurrence of CoNS CRBSI within 90 d and 30 d hospital readmission. Secondary outcomes included hospital length of stay (LOS), 30 d all-cause mortality, and days of Bcx positivity. Groups were compared using Wilcoxon rank-sum for continuous variables and Fisher’s exact for categorical variables using Stata (Version 15).

**Results:**

153 positive CoNS central line Bcx were screened, 40 met study inclusion. 37 received vancomycin as initial abx with a median total tx duration of 13.5 d [IQR 8-14] and 9 d [IQR 6-13.5] after line removal. 22 (55%) received total abx duration of >10 d and 18 (45%) received total abx duration of ≤10 d. Baseline characteristics were similar between >10 d and ≤10 d (table 1). There was no significant difference in primary and secondary outcomes, respectively, between >10 d and ≤10 d (table 2). After catheter removal, there were no significant differences in outcomes whether antibiotics were continued for >7 d (n=18) vs ≤7 d (n=15) (table 3).

**Table 1:**
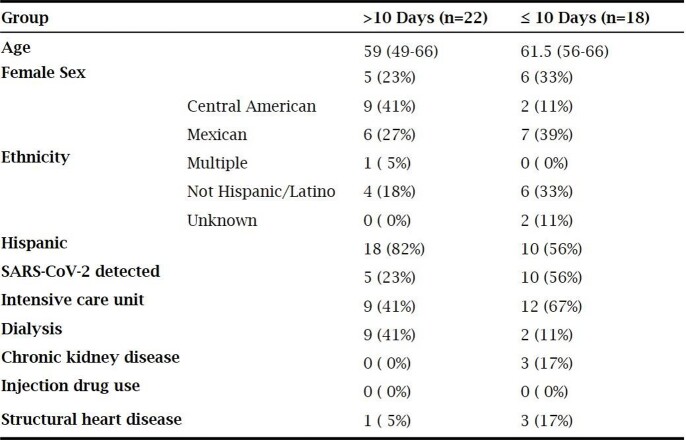
Baseline Characteristics

**Table 2:**
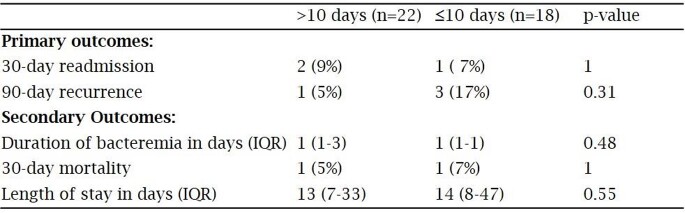
Outcomes according to days of therapy

**Table 3:**
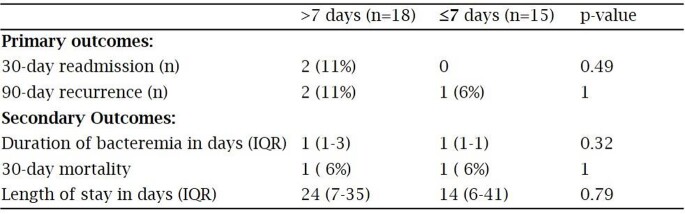
Outcomes post catheter removal days of therapy

**Conclusion:**

Abx tx duration of ≤10 d compared with >10 d for CoNS CRBSI did not result in differences in 90 d recurrence and 30 d readmission. Shorter courses of tx for CoNS CRBSI should be considered as a target for antimicrobial stewardship to minimize antibiotic exposure.

**Disclosures:**

**All Authors**: No reported disclosures.

